# On-Table Versus Deferred Extubation After Paediatric Cardiac Catheterisation Under General Anaesthesia: A Retrospective Cohort Study

**DOI:** 10.3390/jcdd13080388

**Published:** 2026-08-13

**Authors:** Gözde Gürsoy Çirkinoğlu, Halide Hande Şahinkaya, Canan Salman Önemli, Mehmet Ali Efe, Makbule Gürlek, Murat Kaykaç, Mustafa Orhan Bulut, Engin Gerçeker

**Affiliations:** 1 Department of Anesthesiology and Reanimation, Izmir City Hospital, 35540 Izmir, Türkiye; drhhande@yahoo.com (H.H.Ş.); canan.ege.akdeniz@gmail.com (C.S.Ö.); mefe4590@gmail.com (M.A.E.); makbulegurlek@gmail.com (M.G.); drmuratkaykac@gmail.com (M.K.); 2 Department of Pediatric Cardiology, Izmir City Hospital, 35540 Izmir, Türkiye; morhan.bulut@gmail.com (M.O.B.); engingerceker83@gmail.com (E.G.)

**Keywords:** paediatric anaesthesia, cardiac catheterisation, congenital heart disease, on-table extubation, deferred extubation, CRISP score, respiratory complications

## Abstract

Purpose: Extubation timing after paediatric cardiac catheterisation under general anaesthesia remains a challenging clinical decision, particularly in children with cyanotic or haemodynamically significant congenital heart disease. This study aimed to evaluate factors associated with non-on-table extubation and to assess early postoperative respiratory outcomes in this high-risk population. Design: This was a single-centre retrospective cohort study conducted in a paediatric cardiac catheterisation laboratory. Methods: Paediatric patients with cyanotic or haemodynamically significant/complex congenital heart disease who underwent cardiac catheterisation under general anaesthesia with endotracheal intubation were included. Patients were grouped according to whether they were extubated on-table in the catheterisation laboratory or transferred to the intensive care unit with ongoing invasive mechanical ventilation. The primary outcome was non-on-table extubation. Secondary outcomes included extubation timing, reintubation within 48 h, major respiratory complications within 48 h, intensive care unit length of stay, hospital length of stay, and 7-day and 30-day mortality. Logistic regression analysis was used to identify factors associated with non-on-table extubation. Findings: Seventy-two patients were included. On-table extubation was performed in 52 patients (72.2%), whereas 20 patients (27.8%) were not extubated on-table. Patients not extubated on-table were younger, had lower body weight, higher American Society of Anesthesiologists physical status IV (ASA IV) frequency, higher Catheterization Risk Score for Pediatrics (CRISP) scores, lower baseline SpO_2_, and were more frequently undergoing emergency procedures. Reintubation within 48 h occurred only in the non-on-table extubation group (15.0% vs. 0.0%; *p* = 0.019). Major respiratory complications within 48 h were more frequent in patients not extubated on-table (20.0% vs. 3.8%; *p* = 0.047). Intensive care unit and hospital length of stay were also longer in this group. In multivariable analysis, higher CRISP score (adjusted odds ratio 1.22; 95% confidence interval 1.009–1.476; *p* = 0.040) and emergency procedure (adjusted odds ratio 8.74; 95% confidence interval 1.365–55.927; *p* = 0.022) were independently associated with non-on-table extubation. No 7-day mortality occurred in either group. Conclusions: On-table extubation after paediatric cardiac catheterisation under general anaesthesia was feasible in most selected patients with cyanotic or haemodynamically significant/complex congenital heart disease. Higher CRISP score and emergency procedures were independently associated with non-on-table extubation. These findings suggest that catheterisation-specific risk assessment may help anticipate postoperative ventilatory requirements in high-risk paediatric cardiac catheterisation patients.

## 1. Introduction

Congenital heart diseases are among the most common congenital anomalies encountered at birth [1]. Pediatric cardiac catheterization laboratories play a crucial role in both the diagnosis and treatment of these conditions [2]. Procedures performed in these units provide important diagnostic information and therapeutic options, and advances in technology have allowed an increasing number of complex diagnostic and interventional procedures to be performed in pediatric patients.

The complex cardiac anatomy and altered physiology of children with congenital heart disease pose important challenges for anesthetic management. Therefore, analgesia and anesthesia strategies in this population require careful planning and should be delivered by experienced multidisciplinary teams. In pediatric cardiac catheterization laboratories, anesthetic approaches may range from monitored anesthesia care to general anesthesia, depending on the patient’s age, the type and complexity of the procedure, and patient-specific physiological factors [2]. The choice of sedative or anesthetic agents, as well as airway management, should be individualized after a detailed preprocedural assessment, particularly in patients with abnormal circulatory physiology and limited cardiopulmonary reserve [3]. Preprocedural echocardiography is an essential component of the assessment of children undergoing cardiac catheterisation, providing important information regarding cardiac anatomy, ventricular function, and haemodynamic status relevant to procedural and anaesthetic planning.

In procedures performed under general anesthesia with endotracheal intubation, postoperative extubation planning may be challenging because of the underlying altered physiology and the potential risk of respiratory or hemodynamic instability [4]. Although on-table extubation has been reported as feasible in selected pediatric cardiac surgical patients [5,6,7,8,9], evidence specifically addressing extubation timing after pediatric cardiac catheterisation remains limited. In this setting, identifying factors associated with non-on-table extubation may help clinicians better anticipate postoperative ventilatory requirements and early respiratory outcomes.

In the present study, we evaluated pediatric patients with complex cardiac anomalies who underwent cardiac catheterization under general anesthesia in a pediatric cardiac catheterization laboratory. The primary outcome was non-on-table extubation, defined as transfer to the intensive care unit with ongoing invasive mechanical ventilation after completion of the catheterisation procedure. The secondary outcomes included extubation timing, reintubation within the first 48 h, major respiratory complications within the first 48 h, intensive care unit length of stay, hospital length of stay, 7-day mortality, and 30-day mortality. We aimed to compare the clinical and procedural characteristics of patients who were extubated on-table with those who were not, and to evaluate early postoperative respiratory outcomes in these groups.

## 2. Methods

### 2.1. Study Design and Ethical Approval

This single-center, retrospective cohort study was conducted in the pediatric cardiac catheterization laboratory of İzmir City Hospital. The medical records of pediatric patients who underwent diagnostic or interventional cardiac catheterization under general anesthesia between 1 January 2024 and 1 January 2026 were retrospectively reviewed.

The study protocol was approved by the Institutional Ethics Committee of İzmir City Hospital (approval number: 2026/92; date of approval: 4 February 2026). Due to the retrospective design of the study, the requirement for informed consent was waived by the ethics committee. The study was conducted in accordance with the principles of the Declaration of Helsinki. All patient data were anonymized before analysis.

### 2.2. Patient Population

Patients aged 0–18 years who underwent pediatric cardiac catheterization under general anesthesia with endotracheal intubation were screened for eligibility. The study population consisted of children with cyanotic or hemodynamically significant/complex congenital heart disease. Hemodynamically significant or complex disease was defined as the presence of duct-dependent pulmonary or systemic circulation, single-ventricle physiology, significant ventricular outflow tract obstruction, severe valvular stenosis, aortic coarctation associated with impaired cardiac function or significant pressure load, pulmonary hypertension, previous cardiac surgery or palliation, or any clinical condition associated with reduced cardiopulmonary reserve.

Inclusion criteria were as follows: age between 0 and 18 years, diagnosis of cyanotic or hemodynamically significant/complex congenital heart disease, diagnostic or interventional cardiac catheterization performed under general anesthesia, endotracheal intubation performed as part of the anesthetic management in the catheterization laboratory, and availability of data regarding extubation timing and postoperative respiratory outcomes.

Patients undergoing isolated low-risk and hemodynamically stable procedures were not included. These included isolated atrial septal defect closure, isolated patent ductus arteriosus closure, simple shunt lesions without ventricular dysfunction or pulmonary hypertension, and elective diagnostic procedures without clinically relevant hemodynamic impact.

Exclusion criteria were as follows: isolated low-risk congenital cardiac lesions, procedures performed without general anesthesia and endotracheal intubation, patients already intubated before arrival to the catheterization laboratory because of unrelated respiratory or circulatory failure, concomitant surgical procedures during the same session, incomplete data regarding extubation timing or early postoperative respiratory outcomes, and repeated procedures in the same patient. In patients who underwent more than one eligible procedure during the study period, only the first procedure was included.

A total of 72 patients met the inclusion criteria and were included in the final analysis. Patients were categorized according to on-table extubation status as follows: patients extubated in the catheterization laboratory immediately after completion of the procedure were classified as the on-table extubation group, whereas patients transferred to the intensive care unit with ongoing invasive mechanical ventilation were classified as the no on-table extubation group. The decision to perform on-table extubation was not based on a predefined oxygen saturation threshold or a standardized extubation protocol. Extubation readiness was assessed individually by the attending anaesthesiologist based on overall clinical status, including haemodynamic stability, respiratory status, oxygenation relative to baseline physiology, recovery from anaesthesia, and the procedural course.

### 2.3. Data Collection

Data were obtained retrospectively from electronic medical records, anesthesia records, catheterization laboratory records, and intensive care unit follow-up charts. The following variables were recorded: age, sex, body weight, birth history, American Society of Anesthesiologists (ASA) physical status, emergency or elective procedure status, history of previous cardiac surgery, The Catheterization Risk Score for Pediatrics (CRISP) score (CRISP is a pediatric cardiac catheterisation-specific risk stratification tool that incorporates patient-related and procedural factors to estimate the risk associated with cardiac catheterisation [10]), preprocedural inotropic support, primary cardiac diagnosis, procedure type, baseline oxygen saturation, exit oxygen saturation, procedure duration, periprocedural inotropic requirement, extubation timing, reintubation within 48 h, major respiratory complications within 48 h, intensive care unit length of stay, hospital length of stay, 7-day mortality, and 30-day mortality.

Baseline oxygen saturation was recorded while patients were breathing room air. Procedure duration was defined as the time from the start to the completion of the catheterization procedure. Exit oxygen saturation was defined as the oxygen saturation recorded at the end of the procedure before extubation or transfer to the intensive care unit.

Procedure groups were classified according to the expected hemodynamic effect of the intervention as follows: pulmonary blood flow-increasing procedures, pressure load-modifying procedures, diagnostic procedures, and pulmonary blood flow-reducing procedures.

### 2.4. Primary and Secondary Outcomes

The primary outcome was non-on-table extubation. Non-on-table extubation was defined as inability to extubate the patient in the catheterization laboratory immediately after completion of the procedure, requiring transfer to the intensive care unit with ongoing invasive mechanical ventilation.

On-table extubation was defined as extubation performed in the cardiac catheterisation laboratory after completion of the procedure and before transfer from the catheterisation laboratory. No predefined maximum waiting time or minute-based cut-off was used. Extubation readiness was assessed individually by the attending anaesthesiologist based on the patient’s overall clinical condition.

Secondary outcomes included extubation timing, reintubation within the first 48 h, major respiratory complications within the first 48 h, intensive care unit length of stay, hospital length of stay, 7-day mortality, and 30-day mortality.

Extubation timing was categorized as on-table extubation, extubation within the first 6 postoperative hours, or extubation after 6 postoperative hours. Major respiratory complication was defined as the occurrence of at least one of the following within 48 h after the procedure: reintubation, prolonged non-invasive ventilatory support or high-flow nasal cannula requirement for more than 24 h, severe hypoxemia requiring clinical intervention, hypercapnia requiring initiation or escalation of ventilatory support, or clinically significant cyanotic spell requiring active medical treatment. A clinically significant cyanotic spell was defined as an acute worsening of cyanosis and oxygen desaturation relative to the patient’s baseline physiology that required active medical intervention, including escalation of oxygen or ventilatory support, sedation, fluid administration, or pharmacological treatment.

Severe hypoxemia was defined as SpO_2_ < 80% or a clinically significant decrease from the patient’s baseline oxygen saturation requiring escalation of oxygen therapy, ventilatory support, sedation, fluid therapy, or pharmacological intervention.

### 2.5. Statistical Analysis

Statistical analyses were performed using IBM SPSS Statistics for Windows, version 30.0.0.0 (IBM Corp., Armonk, NY, USA). Because of the retrospective design, no formal a priori sample size calculation was performed. All eligible patients during the study period were included. Continuous variables were summarized as median [interquartile range], and categorical variables were summarized as frequencies and percentages. Continuous variables were compared between patients with and without on-table extubation using the Mann–Whitney U test. Categorical variables were compared using the chi-square test or Fisher’s exact test, as appropriate. Because of small cell counts, ASA physical status was dichotomized as ASA IV versus ASA II–III for comparative analyses. Univariable logistic regression analysis was performed to evaluate factors associated with non-on-table extubation. Candidate variables included body weight, CRISP score, ASA IV status, emergency procedure, baseline SpO_2_, exit SpO_2_, and procedure duration. Variables selected a priori based on clinical relevance were entered into the multivariable logistic regression model. The final multivariable model included CRISP score, emergency procedure, and baseline SpO_2_. CRISP score was entered into the logistic regression analysis as a continuous variable; therefore, the corresponding odds ratio represents the change in the odds of non-on-table extubation associated with each one-point increase in CRISP score. Results were reported as odds ratios with 95% confidence intervals. A two-sided *p* value < 0.05 was considered statistically significant. Available-case analysis was used for variables with missing data.

## 3. Results

Baseline and preprocedural characteristics are summarized in Table 1. Compared with patients extubated on-table, those who were not extubated on-table were younger, had lower body weight, were more frequently classified as ASA IV, underwent emergency procedures more often, had higher CRISP scores, and had lower baseline SpO_2_ values. No significant between-group differences were observed in sex, prematurity, previous cardiac surgery, or preprocedural inotropic support.

The lowest baseline SpO_2_ recorded in the overall cohort was 70%; the minimum values were 70% in the on-table extubation group and 80% in the no on-table extubation group.

Cardiac diagnoses and physiological characteristics are presented in Table 2. The overall distribution of primary cardiac diagnosis groups did not differ significantly between the groups. Six patients (8.3%) had single-ventricle physiology; all had previously undergone bidirectional Glenn palliation and were awaiting Fontan completion. Five were extubated on-table and one was not, with no significant between-group difference.

Procedural characteristics are shown in Table 3. Procedure group distribution and procedure duration were similar between the groups. Exit SpO_2_ was lower in patients who were not extubated on-table, whereas periprocedural inotropic requirement did not differ significantly between the groups.

Individual clinical and procedural characteristics of patients who were not extubated on-table are provided in Supplementary Table S1.

Postoperative outcomes are presented in Table 4. Among the 20 patients who were not extubated on-table, 8 (40.0%) were extubated within the first 6 postoperative hours and 12 (60.0%) remained intubated for more than 6 h. Reintubation within 48 h occurred only in the no on-table extubation group. Major respiratory complications within 48 h were also more frequent in this group, and both ICU and hospital length of stay were longer. No 7-day mortality occurred in either group. One 30-day death occurred in the no on-table extubation group, with no statistically significant between-group difference in 30-day mortality.

No 7-day mortality was observed. One patient died within 30 days, and this patient was in the no on-table extubation group; however, the difference in 30-day mortality was not statistically significant (5.0% vs. 0.0%, *p* = 0.278).

Logistic regression results are shown in Table 5. In the multivariable model, each one-point increase in CRISP score was associated with higher odds of non-on-table extubation (adjusted OR 1.22, 95% CI 1.009–1.476; *p* = 0.040), and emergency procedure was also independently associated with the outcome (adjusted OR 8.74, 95% CI 1.365–55.927; *p* = 0.022), whereas baseline SpO_2_ was not independently associated with non-on-table extubation.

## 4. Discussion

In this single-center retrospective cohort study, we evaluated on-table extubation and perioperative factors associated with non-on-table extubation in pediatric patients with cyanotic or hemodynamically significant/complex congenital heart disease undergoing cardiac catheterization under general anesthesia. On-table extubation was feasible in the majority of patients, whereas patients who remained intubated represented a clinically more vulnerable population. Higher CRISP score and emergency procedure were independently associated with non-on-table extubation.

The decision to extubate pediatric cardiac patients after cardiac catheterization is multifactorial and depends on overall clinical readiness rather than any single physiological threshold. In our cohort, 72.2% of patients were extubated on-table. Patients who remained intubated were generally younger, smaller, and clinically higher risk, suggesting that non-on-table extubation primarily reflected underlying vulnerability and perceived lack of readiness for safe extubation.

Previous studies of pediatric cardiac surgery have generally associated on-table or early extubation with shorter ICU and hospital stays and low reintubation rates in appropriately selected patients [5,6,7,8,9]. However, these findings should be interpreted cautiously because most available data are observational and subject to selection bias.

These surgical data cannot be directly extrapolated to the cardiac catheterization laboratory, where procedural stress and haemodynamic changes differ substantially from those associated with cardiopulmonary bypass and open cardiac surgery. To our knowledge, data specifically addressing extubation timing after pediatric cardiac catheterization under general anesthesia remain limited. Our study contributes to this gap by focusing on a selected high-risk catheterization population rather than low-risk lesions such as isolated ASD or PDA closure.

Consistent with the previous surgical literature, patients who were not extubated on-table in our study had a longer ICU and hospital length of stay. However, this association should be interpreted cautiously. Because this was a retrospective observational study, extubation timing was based on clinical judgment rather than random allocation. Patients were extubated on-table when considered clinically ready by the attending anaesthesiologist, whereas those with concerns regarding overall clinical stability or recovery remained intubated for transfer to the ICU. Therefore, patients who remained intubated were likely to have greater baseline severity, lower cardiopulmonary reserve, and higher procedural risk. In this context, non-on-table extubation may be considered a marker of clinical vulnerability rather than a direct cause of prolonged hospitalization. Accordingly, the observed differences in length of stay should not be interpreted as a causal effect of delayed extubation.

The Catheterization Risk Score for Pediatrics (CRISP) is a pediatric cardiac catheterization risk stratification tool developed to estimate the risk of serious adverse events during or after catheterization procedures. It incorporates patient- and procedure-related factors, including age, weight, underlying cardiac diagnosis, hemodynamic vulnerability, and procedural complexity [10]. As a catheterization-specific risk stratification tool incorporating both patient- and procedure-related factors, the CRISP score reflects overall clinical and procedural vulnerability [10]. In our multivariable analysis, each one-point increase in CRISP score was independently associated with increased odds of non-on-table extubation. This finding suggests that CRISP score may be useful not only for procedural risk estimation but also for anticipating postoperative ventilatory requirements after pediatric cardiac catheterization.

Emergency procedures were also independently associated with non-on-table extubation. Emergency procedures are more likely to involve acute or unstable physiology and limited opportunity for preprocedural optimization, which may reduce the likelihood of immediate postoperative extubation. Although the confidence interval was wide because of the small number of emergency cases, procedure urgency remained independently associated with non-on-table extubation. However, the association between emergency procedures and non-on-table extubation may also have been influenced by procedural timing and the availability of time, staffing, or resources during out-of-office hours, which could not be evaluated retrospectively.

Baseline SpO_2_ was lower in patients who were not extubated on-table and was significant in univariable analysis, but it did not remain independently associated with non-on-table extubation after adjustment. Importantly, extubation decisions were not based on a predefined SpO_2_ threshold. Oxygen saturation was interpreted relative to each patient’s underlying physiology and together with haemodynamic stability, respiratory status, recovery from anaesthesia, and the procedural course. Although baseline and exit SpO_2_ values were lower in patients who were not extubated on-table, neither provided an independent basis for the extubation decision. These findings suggest that oxygen saturation should be interpreted within the broader clinical and hemodynamic context rather than used as an isolated decision-making criterion.

Early respiratory outcomes also differed between groups. Major respiratory complications within the first 48 h and reintubation within 48 h were more frequent among patients who were not extubated on-table. These differences most likely reflect the greater baseline severity of patients considered unsuitable for on-table extubation rather than a harmful effect of delayed extubation itself. Importantly, no patient extubated on-table required reintubation within the first 48 h, suggesting that on-table extubation was safe in appropriately selected patients in our cohort.

This study has several limitations. First, it was a single-center retrospective study with a relatively small sample size, particularly in the no on-table extubation and delayed extubation subgroups. Second, extubation decisions were based on clinician judgment rather than a standardized extubation protocol, which may have introduced selection bias and confounding by indication. Furthermore, procedural timing and potential out-of-hours staffing effects on extubation decisions were not systematically recorded and therefore could not be evaluated retrospectively. Third, the study population was intentionally restricted to patients with cyanotic or hemodynamically significant/complex congenital heart disease; therefore, the findings may not be generalizable to low-risk pediatric catheterization populations. Fourth, because of the limited number of adverse events, some outcomes could only be analyzed descriptively or with limited statistical power. Finally, unmeasured factors such as detailed intraoperative ventilation parameters, blood gas values, anesthetic drug doses, or clinician-specific decision-making may have influenced extubation timing.

In conclusion, on-table extubation after pediatric cardiac catheterization under general anesthesia was feasible in the majority of selected patients with cyanotic or hemodynamically significant/complex congenital heart disease. Patients who were not extubated on-table represented a clinically higher-risk population, and a higher CRISP score and more emergency procedures were independently associated with non-on-table extubation. These findings suggest that CRISP score and procedure urgency may help clinicians anticipate the need for postoperative ventilatory support in high-risk pediatric cardiac catheterization patients. Prospective studies using standardized extubation criteria are needed to validate these findings and to develop risk-adapted extubation strategies in this population.

## Figures and Tables

**Table 1:** Baseline and preprocedural characteristics according to on-table extubation status.

Variable	Overall Cohort *n* = 72	On-Table Extubation *n* = 52	No On-Table Extubation *n* = 20	*p* Value
Age, months	12.0 [5.0–70.5]	24.0 [9.54–84.0]	6.0 [2.17–11.75]	0.005
Weight, kg	8.00 [4.63–17.25]	11.50 [6.29–23.75]	3.96 [3.26–6.43]	<0.001
Male sex	43 (59.7%)	31 (59.6%)	12 (60.0%)	0.976
Prematurity	22 (30.6%)	15 (28.8%)	7 (35.0%)	0.612
ASA IV	15 (20.8%)	6 (11.5%)	9 (45.0%)	0.003
Emergency procedure	8 (11.1%)	2 (3.8%)	6 (30.0%)	0.005
Previous cardiac surgery				0.757
No previous surgery	31 (43.1%)	23 (44.2%)	8 (40.0%)	
Previous surgery < 30 days	2 (2.8%)	1 (1.9%)	1 (5.0%)	
Previous surgery > 30 days	39 (54.2%)	28 (53.8%)	11 (55.0%)	
Preprocedural inotropic support	2 (2.8%)	1 (1.9%)	1 (5.0%)	0.481
CRISP score	7.0 [4.0–9.0]	6.0 [3.25–8.0]	9.0 [7.25–11.0]	0.001
Baseline SpO_2_, %	96.0 [92.0–98.0]	96.0 [94.0–99.0]	92.5 [91.25–96.0]	0.002

Data are presented as median [interquartile range] or *n* (%). Mann–Whitney U test was used for continuous variables. Categorical variables were compared using the chi-square test or Fisher’s exact test, as appropriate. ASA physical status was dichotomized as ASA IV versus ASA II–III because of small cell counts. Baseline SpO_2_ was recorded on room air in all patients. ASA: American Society of Anesthesiologists; CRISP: Catheterization Risk Score for Pediatrics; SpO_2_: peripheral oxygen saturation.

**Table 2:** Cardiac diagnoses and physiological characteristics according to on-table extubation status.

Variable	Overall Cohort *n* = 72	On-Table Extubation *n* = 52	No On-Table Extubation *n* = 20	*p* Value
Primary cardiac diagnosis group				0.923
Left-sided obstructive lesions	17 (23.6%)	13 (25.0%)	4 (20.0%)	
Pulmonary stenosis/RVOT obstruction	14 (19.4%)	9 (17.3%)	5 (25.0%)	
Shunt lesions/pulmonary hypertension	11 (15.3%)	9 (17.3%)	2 (10.0%)	
Tetralogy of Fallot/pulmonary atresia/MAPCA spectrum	8 (11.1%)	6 (11.5%)	2 (10.0%)	
Transposition of the great arteries spectrum	7 (9.7%)	4 (7.7%)	3 (15.0%)	
Single-ventricle/hypoplastic ventricle lesions	6 (8.3%)	5 (9.6%)	1 (5.0%)	
Double-outlet right ventricle	5 (6.9%)	3 (5.8%)	2 (10.0%)	
Other complex congenital heart disease	4 (5.6%)	3 (5.8%)	1 (5.0%)	
Physiological characteristics				
Cyanotic congenital heart disease	23 (31.9%)	15 (28.8%)	8 (40.0%)	0.405
Single-ventricle physiology	6 (8.3%)	5 (9.6%)	1 (5.0%)	1.000
Pre-Glenn single-ventricle physiology	0 (0.0%)	0 (0.0%)	0 (0.0%)	—
Post-Glenn/pre-Fontan physiology	6 (8.3%)	5 (9.6%)	1 (5.0%)	1.000

Data are presented as *n* (%). Primary cardiac diagnoses were grouped according to the predominant anatomical and physiological lesion. The *p* value for the overall distribution of primary cardiac diagnosis groups was calculated using the Fisher–Freeman–Halton exact test because of small expected cell counts. Physiological characteristics were compared between groups using Fisher’s exact test, as appropriate. Cyanotic congenital heart disease included transposition of the great arteries, tetralogy of Fallot, pulmonary atresia spectrum, critical pulmonary stenosis, and single-ventricle/hypoplastic ventricle physiology associated with cyanotic circulation. All patients with single-ventricle physiology had previously undergone bidirectional Glenn palliation and were awaiting Fontan completion; therefore, no pre-Glenn single-ventricle patients were included in the cohort. RVOT: right ventricular outflow tract; MAPCA: major aortopulmonary collateral artery; CHD: congenital heart disease.

**Table 3:** Procedural characteristics according to on-table extubation status.

Variable	Overall Cohort *n* = 72	On-Table Extubation *n* = 52	No On-Table Extubation *n* = 20	*p* Value
Procedure group				0.497
Pulmonary blood flow-increasing procedures	18 (25.0%)	15 (28.8%)	3 (15.0%)	
Pressure load-modifying procedures	33 (45.8%)	24 (46.2%)	9 (45.0%)	
Diagnostic procedures	19 (26.4%)	12 (23.1%)	7 (35.0%)	
Pulmonary blood flow-reducing procedures	2 (2.8%)	1 (1.9%)	1 (5.0%)	
Procedure duration, min	140.0 [110.0–160.0]	140.0 [105.0–163.75]	137.5 [115.0–153.75]	0.890
Exit SpO_2_, %	97.0 [95.0–99.0]	98.0 [95.0–99.0]	95.0 [94.0–97.5]	0.007
Periprocedural inotropic requirement	4 (5.6%)	2 (3.8%)	2 (10.0%)	0.307

Data are presented as median [interquartile range] or *n* (%). Mann–Whitney U test was used for continuous variables. Categorical variables were compared using the chi-square test or Fisher’s exact test, as appropriate. The *p* value for procedure group refers to the overall comparison across procedure categories. SpO_2_: peripheral oxygen saturation.

**Table 4:** Postoperative outcomes according to on-table extubation status.

Outcome	Overall Cohort *n* = 72	On-Table Extubation *n* = 52	No on-Table Extubation *n* = 20	*p* Value
Reintubation within 48 h	3 (4.2%)	0 (0.0%)	3 (15.0%)	0.019
Major respiratory complication within 48 h	6 (8.3%)	2 (3.8%)	4 (20.0%)	0.047
ICU length of stay, days	1.0 [1.0–4.0]	1.0 [1.0–2.0]	3.5 [1.0–14.0]	0.001
Hospital length of stay, days	3.0 [2.0–8.25]	3.0 [2.0–4.5]	16.0 [3.0–30.0]	0.001
7-day mortality	0 (0.0%)	0 (0.0%)	0 (0.0%)	—
30-day mortality	1 (1.4%)	0 (0.0%)	1 (5.0%)	0.278

Data are presented as median [interquartile range] or *n* (%). Mann–Whitney U test was used for continuous variables. Categorical variables were compared using the chi-square test or Fisher’s exact test, as appropriate. Extubation timing categories are presented descriptively because on-table extubation status defined the study groups. ICU: intensive care unit. In univariable logistic regression analysis, higher CRISP score, ASA IV status, emergency procedure, and lower baseline SpO_2_ were associated with non-on-table extubation. In the multivariable model including CRISP score, emergency procedure, and baseline SpO_2_, both CRISP score and emergency procedure remained independently associated with non-on-table extubation. Each one-point increase in CRISP score was associated with increased odds of non-on-table extubation (adjusted OR 1.22, 95% CI 1.009–1.476, *p* = 0.040). Emergency procedures were also independently associated with non-on-table extubation (adjusted OR 8.74, 95% CI 1.365–55.927, *p* = 0.022). Baseline SpO_2_ was not independently associated with the outcome after adjustment (adjusted OR 0.943, 95% CI 0.843–1.054, *p* = 0.298).

**Table 5:** Logistic regression analysis for non-on-table extubation.

Variable	Unadjusted OR	95% CI	*p* Value	Adjusted OR	95% CI	*p* Value
Weight, kg	0.976	0.940–1.015	0.222	—	—	—
CRISP score	1.29	1.08–1.54	0.005	1.22	1.009–1.476	0.040
ASA IV	6.27	1.84–21.35	0.003	—	—	—
Emergency procedure	10.71	1.95–59.03	0.006	8.74	1.365–55.927	0.022
Baseline SpO_2_, %	0.889	0.798–0.990	0.034	0.943	0.843–1.054	0.298
Exit SpO_2_, %	0.887	0.767–1.025	0.104	—	—	—
Procedure duration, min	0.999	0.983–1.015	0.898	—	—	—

## Data Availability

The data presented in this study are available from the corresponding author upon reasonable request.

## Supplementary Materials

The following supporting information can be downloaded at: https://www.mdpi.com/article/10.3390/jcdd13080388/s1, Table S1. Individual clinical and procedural characteristics of patients not extubated on-table.
