# Supplementary material for: On-Table Versus Deferred Extubation After Paediatric Cardiac Catheterisation Under General Anaesthesia: A Retrospective Cohort Study
Source: J Cardiovasc Dev Dis. 2026 Aug 13;13(8):388. doi: 10.3390/jcdd13080388 (PMC13513607; doi:10.3390/jcdd13080388)
Supplement: Supplementary file 1 [file jcdd-13-00388-s001.zip › jcdd-4404434-supplementary.pdf]

Table S1. Individual clinical and procedural characteristics of patients not extubated on-table.

Patient	Primary cardiac diagnosis	Procedure group	ASA PS	Emergency procedure	CRISP score	Single-ventricle physiology/stage	Baseline SpO_2 (%)	Exit SpO_2 (%)	Preprocedural inotropic support	Periprocedural inotropic requirement	Extubation timing
1	Coarctation of the aorta	Diagnostic	III	Yes	11	No	92	95	No	No	>6 h
2	Aortic stenosis with ASD	Pressure load-modifying	IV	No	10	No	96	97	No	No	>6 h
3	Aortic transection	Pressure load-modifying	III	No	6	No	98	98	No	No	>6 h
4	Aortic stenosis	Pressure load-modifying	III	No	10	No	99	99	No	No	≤6 h
5	Double-outlet right ventricle	Diagnostic	III	No	3	No	92	94	No	No	≤6 h
6	Double-outlet right ventricle	Diagnostic	IV	No	12	No	80	84	No	No	≤6 h
7	d-Transposition of the great arteries	Diagnostic	III	No	7	No	91	94	No	No	≤6 h
8	d-Transposition of the great arteries	Pulmonary blood flow-increasing	III	No	13	No	92	94	No	No	>6 h

9	Hypoplastic left ventricle	Diagnostic	IV	No	9	Yes; post-Glenn/pre-Fontan	92	95	No	Yes	>6 h
10	Pulmonary atresia with intact ventricular septum	Pressure load-modifying	IV	Yes	12	No	88	90	No	No	≤6 h
11	Critical pulmonary stenosis with PDA	Pulmonary blood flow-increasing	III	Yes	9	No	96	98	No	No	≤6 h
12	Pulmonary stenosis	Diagnostic	IV	Yes	10	No	92	94	No	No	≤6 h
13	Pulmonary stenosis	Pressure load-modifying	III	No	9	No	94	95	No	Yes	>6 h
14	Pulmonary balloon valvuloplasty	Pressure load-modifying	III	Yes	11	No	94	96	No	No	>6 h
15	Pulmonary stenosis	Pressure load-modifying	IV	No	9	No	94	97	No	No	>6 h
16	Secundum ASD with PDA	Pulmonary blood flow-increasing	IV	No	8	No	93	95	No	No	>6 h
17	Transposition of the great arteries	Diagnostic	III	No	6	No	96	98	No	No	≤6 h

18	Ventricular septal defect	Pressure load-modifying	IV	No	5	No	98	99	No	No	>6 h
19	Ventricular septal defect with tetralogy of Fallot	Pressure load-modifying	IV	No	8	No	85	89	Yes	No	>6 h
20	Ventricular septal defect with pulmonary atresia	Pulmonary blood flow-reducing	III	Yes	11	No	80	95	No	No	>6 h

Footnote: Data are presented individually for the 20 patients who were transferred to the intensive care unit with ongoing invasive mechanical ventilation after completion of the catheterisation procedure. Baseline SpO_2 was measured on room air before the procedure, whereas exit SpO_2 was recorded at the end of the procedure before transfer to the intensive care unit. One patient had single-ventricle physiology and had previously undergone bidirectional Glenn palliation and was awaiting Fontan completion. No pre-Glenn single-ventricle patients were included. Eight patients (40.0%) were extubated within the first 6 postoperative hours, whereas 12 patients (60.0%) remained intubated for more than 6 hours. Extubation decisions were individualized and were not based on a predefined SpO_2 threshold. ASA PS: American Society of Anesthesiologists Physical Status; CRISP: Catheterization Risk Score for Pediatrics; SpO_2: peripheral oxygen saturation; ASD: atrial septal defect; PDA: patent ductus arteriosus.
